# Molecular detection and identification of *Plasmodium* spp. isolated from captive-bred cynomolgus monkeys in Bogor, Indonesia

**DOI:** 10.14202/vetworld.2024.337-343

**Published:** 2024-02-08

**Authors:** Uus Saepuloh, Lis Rosmanah, Risqa Novita, Ellis Dwi Ayuningsih, Susi Soviana, Upik Kesumawati Hadi, Huda Shalahudin Darusman

**Affiliations:** 1 Primate Research Center, Bogor Agricultural University, Jl. Lodaya II/5, Bogor, 16151, Indonesia; 2 Research Center for Pharmaceutical Ingredients and Traditional Medicine, National Research and Innovation Agency (BRIN), Genomic Building, Cibinong Science Center, Jl. Raya Bogor No. 490, Cibinong, 16915 Indonesia; 3 Primatology Study Program, Graduate School of IPB University, Jl. Lodaya II/5, Bogor, 16151, Indonesia; 4 Department of Animal Infectious Diseases and Veterinary Public Health, Faculty of Veterinary Medicine, Bogor Agricultural University, Jl. Agatis, Dramaga, Bogor, 16680, Indonesia

**Keywords:** malaria, phylogenetic tree, *Plasmodium inui*, small subunit ribosomal RNA

## Abstract

**Background and Aim:**

Asian macaques are natural hosts of several *Plasmodium* species. Some monkey malaria parasites may infect humans and cause zoonotic infections. This study was conducted to estimate the prevalence of monkey malaria parasites in Bogor, Indonesia, based on molecular detection and identification, particularly in cynomolgus monkeys, which have a wide geographic distribution and share extensive habitats with humans. These data are needed to evaluate the status of simian malaria among macaques in Bogor and to study the potential risks to human health. These updated data will provide sufficient information for implementing malaria control strategies in the future and for developing a potential malaria vaccine using monkeys as an animal model.

**Materials and Methods:**

Blood samples of 274 cynomolgus monkeys (*Macaca fascicularis*) were collected and identified using microscopy. DNA was extracted from positive blood samples and analyzed using polymerase chain reaction (PCR) to amplify the small subunit ribosomal RNA (*SSU rRNA*) target gene using consensus primers for *Plasmodium* species. The PCR-positive samples were then nucleotide-sequenced using commercial sequencing services, analyzed using the BioEdit program, and aligned using Basic Local Alignment Search Tool from the National Center for Biotechnology Information. Phylogenetic trees were constructed using MEGA 11.0 and the neighbor-joining (NJ) method to determine the kinship of *Plasmodium*. Bootstrapping was performed using 500 replicates to assess the robustness of tree topologies.

**Results:**

Thirty-eight of the 274 microscopically positive samples for *Plasmodium* spp. were also positive using PCR, resulting in a 1640 bp amplicon. Further, analysis using nucleotide sequencing confirmed that these positive samples were *Plasmodium inui* with more than 99% nucleotide identity compared to GenBank sequences. Phylogenetic tree analysis of the *SSU rRNA* partial gene showed that all our isolates clustered and were closely related to a *P. inui* strain isolated from cynomolgus macaques in South China in 2011.

**Conclusion:**

*P. inui* is the predominant malaria parasite in cynomolgus monkeys from Bogor.

## Introduction

Indonesia is one of nine malaria-endemic countries in Southeast Asia, accounting for 21% of reported cases and 16% of malaria-related deaths [1]. All four species of human malaria parasites are found in Indonesia. *Plasmodium vivax* was the predominant species, except in Papua, where *Plasmodium* *falciparum* was the dominant species. *Plasmodium malariae* and *Plasmodium* *ovale* are mostly found in eastern Indonesia, Nusa Tenggara Timur, and Papua. *Plasmodium* *knowlesi*, a common simian *Plasmodium* that naturally infects macaque monkeys in Southeast Asia, has recently been reported to infect humans in Kalimantan [2]. Understanding the diversity and distribution of non-human primate malaria is an important first step in predicting the potential zoonotic risk of non-human primate malaria. Studying this diversity can provide critical insights into our understanding of human malaria, as several human malarial species result from host switching from non-human primates [3]. The zoonotic spillover of wild primate malaria is an emerging global public health concern. More than 30 species of *Plasmodium* have been reported in non-human primates, such as apes, gibbons, New World monkeys, and Old World monkeys.

The cynomolgus monkey is a long-tailed macaque living in a wide variety of habitats, such as primary lowland rainforests, disturbed and secondary rainforests, shrublands, rivers, and coastal forests. The native range of this species includes most of mainland Southeast Asia, from southeastern Bangladesh through Malaysia; Maritime Southeast Asia islands of Sumatra, Java, and Borneo; offshore islands; the islands of the Philippines; and the Nicobar Islands in the Bay of Bengal. A high rate of tropical deforestation, thriving wildlife trade and hunting networks, and an increasing human population have reduced the natural habitat of monkeys and increased the risk of zoonotic diseases. Long-tailed macaques are a natural host of five *Plasmodium* species: *P. knowlesi*, *Plasmodium cynomolgi*, *Plasmodium coatneyi*, *Plasmodium fieldi*, and *Plasmodium inui* [4, 5]. Human diseases caused by *P. knowlesi* transmission have been reported in the Malaysian Peninsula, Southern Thailand, and Borneo, Indonesia. *P. inui* and *P. cynomolgi* have been implicated in human infection under both experimental and accidental conditions.

Despite its importance and potential hazards to human health, epidemiological studies that have evaluated the status of malaria among macaques in Indonesia, especially in Bogor, West Java, are lacking. This study was conducted based on molecular detection and identification to estimate the prevalence of monkey malaria parasites, particularly in cynomolgus monkeys, in Bogor, Indonesia. These data are needed to evaluate the status of simian malaria among macaques in Bogor and to study the potential risks to human health. These updated data will provide sufficient information for implementing malaria control strategies in the future and for developing a potential malaria vaccine using monkeys as an animal model.

## Materials and Methods

### Ethical approval

The Institutional Animal Care and Use Committee of the Primate Research Center (PRC) of the IPB University approved this study (number 20-E005).

### Study period and location

The study was conducted from July 2020 to August 2021 in the ex-situ captive facility at the Primate Research Centre (PRC) of IPB University located at the IPB University Dramaga, Bogor, West Java.

### Sample collection and site

A total of 274 long-tailed monkeys were collected from the captive breeding facilities of the PRC, IPB University, Dramaga, Bogor, Indonesia. Before blood collection, the monkeys were anesthetized with an intramuscular injection of Ketamil® (10% ketamine hydrochloride; Troy Laboratories Pty. Ltd., NSW, Australia). Blood samples (3 mL) were collected from the femoral veins, placed in vacutainer tubes containing ethylenediaminetetraacetic acid as an anticoagulant, and stored at 4°C until further use. The collected blood was divided into two parts. One part was used for Giemsa-stained thick and thin blood smears to detect the *Plasmodium* parasite using a microscope (Eclipse 80i, Nikon, Japan). The other part of the blood, which was previously identified as positive for *Plasmodium* by microscopy, was subjected to DNA extraction for molecular detection and characterization. Genomic DNA was extracted from blood using the QIAamp DNA Blood Mini Kit (Qiagen, Hilden, Germany) according to the manufacturer’s instructions.

### Molecular detection of *Plasmodium*

A partial small subunit ribosomal RNA (*SSU rRNA*) gene was the target gene for polymerase chain reaction (PCR) amplification of malaria *Plasmodium*. The primers used in this PCR were adapted from Singh *et al*. [5]; rPLU1 and rPLU5 targeted to *SSU rRNA* resulted in *Plasmodium* spp. 1640 bp amplicons. PCR amplification was performed using a thermocycler machine (Gene Amp® PCR System 9700, Applied Bio SystemTM, Foster City, CA, USA). Each reaction volume of 25 μL contained 1 μL of 10 pmol/μL of each primer (Integrated DNA Technologies, Singapore), 12.5 μL of Go Taq® Green Master Mix (Promega, Madison, WI, USA), and 2.5 μL of 25–50 ng/μL of DNA templates and was adjusted with nuclease-free water to the total volume of 25 μL. The PCR process was run under the following conditions: pre-denaturation at 94°C for 3 min; 40 cycles of denaturation at 94°C for 30 s, annealing at 56°C for 30 s, extension at 72°C for 30 s; and a final extension at 72°C for 5 min. The PCR products were visualized using 1.8% agarose gel electrophoresis, stained with SYBR Safe DNA Staining (Thermo Scientific, USA), and detected using a Gel Doc 2000 (Bio-Rad, USA) [5, 6].

### Nucleotide sequence analysis

The PCR products were nucleotide-sequenced using the Sanger method at 1^st^ Base, Malaysia, a commercial sequencing provider. All sequence results were edited using BioEdit software version 7.7 (bioedit.software.informer.com/) [7] and aligned using the Basic Local Alignment Search Tool (BLAST) program from National Center for Biotechnology Information, National Institute of Health, USA (https://blast.ncbi.nlm.nih.gov) to determine the identity of our *Plasmodium* isolate in comparison to those of the sequence data in GenBank. Nucleotide sequences were aligned using ClustalW and then adjusted for phylogenetic tree construction using MEGA 11.0 (https://www.megasoftware.net) based on the neighbor-joining method [8]. Genetic distances were estimated using Kimura’s two-parameter method and confidence values were assigned to the tree nodes by bootstrap analysis on 500 replicates. Nucleotide sequences of others *Plasmodium* species from GenBank were used as references: L07559.1 (*P. cynomolgi*), HM032051.1 (*P. inui* isolate South China), EU400386.1 (*P. inui* isolate WPN4), EU400387.1 (*P. inui* isolate WPN5), U72541.1 (*P. inui*), AB287276.1 (*P. inui*), JQ627155.1 (*P. vivax*), M61722.1 (*Plasmodium* *fragile*), AB287280.1 (*Plasmodium* *hylobati*), AB265791.1 (*P. coatneyi*), AB287270.1 (*Plasmodium gonderi*), AB287282.1 (*P. fieldi*), AB287287.1 (*Plasmodium simiovale*), FJ619089.1 (*P. knowlesi*), M54897.1 (*P. malariae*), L48986.1 (*P. ovale*), M19172.1 (*P. falciparum*), and Z25819.1 (*Plasmodium reichenowi*).

## Results

### PCR amplification and sequence analysis of *Plasmodium* spp.

**Figure-1:**
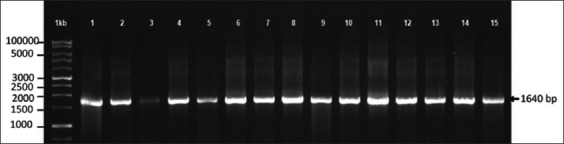
Polymerase chain reaction amplification to the target small subunit ribosomal RNA gene was visualized using 1.8% agarose electrophoresis stained with SYBR Safe and the Gel-Doc machine. A 1640-bp amplicon of the specific target was detected. 1 kb: DNA Ladder, 1-15: number of samples.

A total of 38 of the 274 (13.8%) cynomolgus monkey blood samples tested positive for *Plasmodium* spp. by microscopy. The positivity of these samples was confirmed by PCR amplification that indicated a 1640 bp specific band of *Plasmodium* spp. *SSU rRNA* target gene (Figure-1). Nucleotide sequence analysis using BLAST showed that all isolates had partial *SSU rRNA* of *P. inui* with 98%–99% identities and a query cover percentage of 100%.

### Molecular characterization of *P. inui*

**Figure-2:**
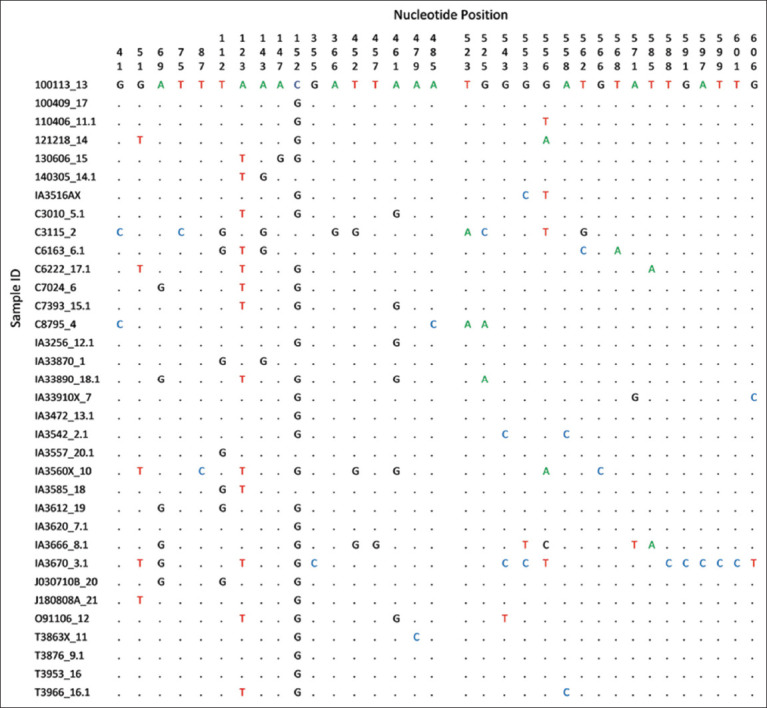
Polymorphisms of 623 partial nucleotide sequences of the small subunit ribosomal RNA gene of *Plasmodium inui* obtained from cynomolgus monkeys (*Macaca fascicularis*) in this study. Nucleotide positions in the genes are numbered above the polymorphic sites; sample IDs are indicated by the tattoo number of the animals; dots show identical nucleotide residues.

Further, sequence analysis was conducted to compare nucleotide variation among the isolates. A total of 34 nucleotide sequences of partial *SSU rRNA* genes with 623 bp contig sequences were analyzed. Figure-2 presents the nucleotide variations in partial *SSU rRNA* genes of the 34 *Plasmodium* spp. isolates. Multiple alignment analysis identified 583/623 (93.6%) conserved sites and 40/623 (6.4%) variable (polymorphic) sites. Variable sites comprised 16 (2.6%) parsimony-informative sites and 24 (3.8%) singleton sites. We calculated the genetic distance of the isolates using the Kimura-2 parameter method [9]: 0.0000–0.0329 (data not shown).

### Phylogenetic tree analysis

**Figure-3:**
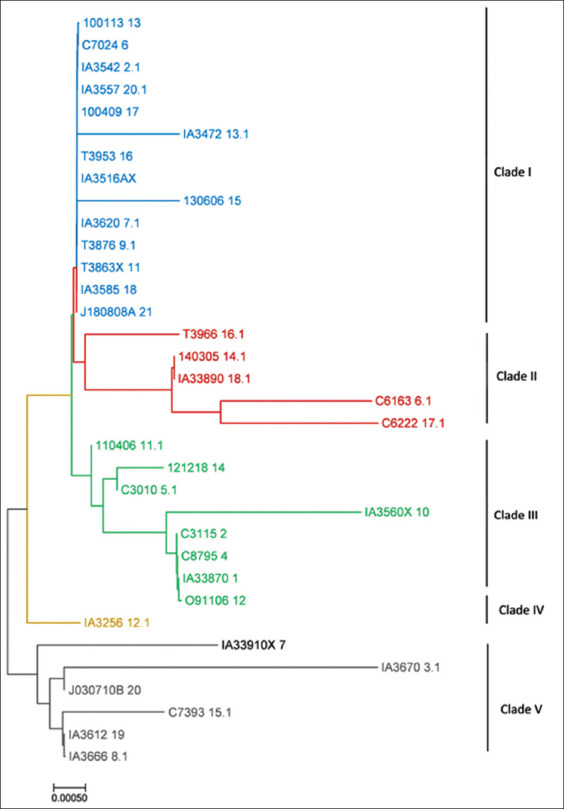
Phylogenetic tree analysis among the *Plasmodium inui* partial gene of the small subunit ribosomal RNA of 34 isolates obtained from Indonesian cynomolgus monkeys. The tree was constructed using the neighbor-joining method.

**Figure-4:**
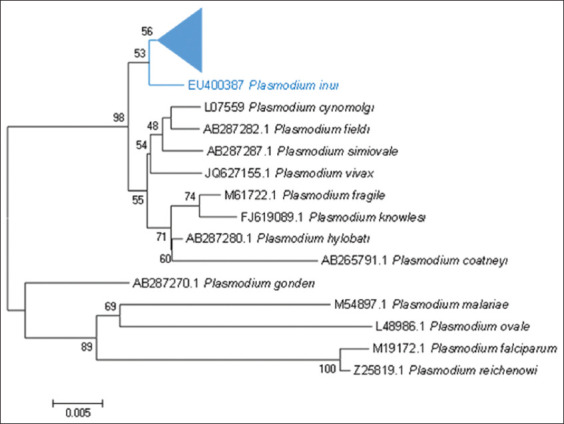
Phylogenetic tree analysis of the *Plasmodium inui* partial gene of the small subunit ribosomal RNA of 34 isolates obtained from Indonesian cynomolgus monkeys (indicated with an arrow) compared with sequences of other *P. inui* as well as with sequences of known species in GenBank. The tree was constructed using the neighbor-joining method.

The results of phylogenetic tree reconstruction showed the relationship among the 34 *Plasmodium* isolates obtained from Indonesian cynomolgus monkeys in the present study (Figure-3). Among the isolates, the polymorphic sites of the *SSU rRNA* gene included five clades (I, II, III, IV, and V). The phylogenetic tree reconstruction of all isolates compared with that of other simian and human *Plasmodium* isolates in GenBank showed that all isolates clustered as *P. inui* (Figure-4). The isolates had the closest relationship with the *P. inui* isolate from South China isolated from *Macaca fascicularis* in South China in 2011 (HM032051) [10] and grouped with other *P. inui* such as *P. inui* isolated from wild *M. fascicularis* from Thailand (EU400387), *Macaca cyclopis* (AB287276), and *Macaca mulatta* (U72541).

## Discussion

In the present study, we report the molecular detection and characterization of *Plasmodium* spp. found in blood samples of cynomolgus monkeys in Bogor, Indonesia, that were determined to be positive by microscopy. Based on BLAST and phylogenetic tree analyses of the partial *SSU rRNA* gene, all isolates were clustered as *P. inui*. Thirty-eight of 274 (13.8%) cynomolgus monkeys evaluated from our macaque breeding facilities were infected with *P. inui*. This infection may have been caused by the competent mosquito vectors present in the area. Some *Anopheles* mosquitoes, such as *Anopheles aconitus*, *Anopheles barbirostris*, *Anopheles nigerrimus*, and *Anopheles subpictus*, have been reported in the area around Dramaga-Bogor [11], possibly transmit malaria. It is also possible that the macaques have already been infected by *Plasmodium* parasite in their original habitat, Sumatra Island, before being transported and bred in our facility in Bogor, West Java.

To evaluate the status of *Plasmodium* parasite and study its potential hazards to human health, we estimated the prevalence of simian malaria in cynomolgus monkeys at our breeding colony facilities. Although the results of the present study do not indicate the prevalence of *P. inui* in the macaque population in Indonesia, we suggest that *P. inui* is the most prevalent simian malaria parasite present in wild populations in Indonesia. The prevalence of *P inui* in wild macaque populations in Thailand and Taiwan can reach more than 20% [12–14].

*P. inui* is the most widely distributed malaria parasite in Old World monkeys and is naturally found in *M. fascicularis*, *Macaca nemestrina*, *Macaca mulatta*, *Macaca radiata*, *M. cyclopis*, *Cynopithecus niger*, *Presbytis obscurus*, and *Pavo cristatus* [15]. Regarding the potential zoonosis of *P. inui* for human health, whether this simian parasite poses a danger of cross-contamination to humans is unknown and requires further investigation in more monkey populations, human populations, and malaria vectors [10]. Although *P. inui* is experimentally transmissible to humans, natural human infections have not yet been reported or documented in Indonesia. The potential for zoonosis is influenced by human habitation and behavior as well as the adaptive capabilities of parasites and vectors [16]. At present, zoonotic *Plasmodium* infections in humans are increasing in Laos [17], Malaysia [18], Thailand [19], Myanmar [20], Philippines [21], Singapore [22], Brunei [23], Cambodia [24], and Vietnam [25], including *P. inui* infections in Malaysia and Peninsular Malaya. The prevalence of *P. inui* was 66.7% in Pahang. *P. inui* has a wide geographical range in Asia. Despite mild clinical symptoms, the high prevalence of *P. inui*, supported by the presence of *Anopheles* mosquitoes, is a risk factor for human infection. *A. aconitus* and *Anopheles vagus*, which are vectors of malaria in primates, have been found in the same location. The prevalence of *P. inui* in *M. fascicularis* warrants investigation of the presence of *P. inui* in *Anopheles* mosquitoes and humans in the same location or within a 1-km radius of *M. fascicularis* to prevent the spread of infection [10].

We used the mitochondrial *SSU rRN*A gene as a molecular marker to identify *Plasmodium* species and constructed a phylogenetic tree for further analysis. The *SSU rRNA* gene has frequently been used as a molecular marker for *Plasmodium* species phylogenetic analysis [16]. Multiple alignments of 34 *P. inui* isolates with 623 bp nucleotide sequences of partial *SSU rRNA* showed a 6.4% variable (polymorphic) site (Figure-2), indicating that this region is appropriate for species determination.

However, this polymorphic site also indicated the presence of a nucleotide variation that may have caused the different clades of *P. inui* in our isolates. The variable region (polymorphic) of this *SSU rRNA* gene suggested five clades (I–V) among the isolates (Figure-3). Polymorphic markers are necessary for studying variations in *P. inui* in populations and may help determine the genetic structure of populations and detect the lineage-specific evolutionary history of human malaria parasites [26].

Figure-4 shows the phylogenetic tree reconstruction of *P. inui* isolated in the present study, including sequences of other simian and human *Plasmodium* species. All isolates were clustered as *P. inui* and were distant from human *Plasmodium*. *P. inui* is closely related to *P. hylobati*, which branches from other simian *Plasmodium* spp. such as *P. cynomolgi*, *P. fieldi*, *P. simiovale*, *P. coatneyi*, *P. fragile*, and *P. knowlesi*. *P. gonderi* branched at the earliest, and human *P. vivax* is closely related to this simian parasite. The phylogenetic tree reconstruction of *Plasmodium* using the *SSU rRNA* gene was consistent with the results obtained using other molecular markers. Therefore, this phylogenetic tree based on *SSU rRNA* is indispensable for positioning new species [27].

## Conclusion

Our findings suggest that *P. inui* is the most prevalent malaria parasite in cynomolgus monkeys present in our captive breeding facility in Bogor. These data are needed to evaluate the status of simian malaria among macaques in Bogor and to study the potential risks to human health. Therefore, further investigations are needed to provide adequate information for the implementation of malaria control strategies in the future, as well as to develop a potential malaria vaccine using monkeys as animal models.

## Authors’ Contributions

US, LR, SS, UKH, and HSD: Designed and supervised the study. LR and RN: Collected samples. US, EDA, LR, and RN: Performed laboratory work and drafted the manuscript. US, RN, SS, UKH, and HSD: Revised the manuscript. All authors have read, reviewed, and approved the final version of the manuscript.
